# rs10865331 Associated with Susceptibility and Disease Severity of Ankylosing Spondylitis in a Taiwanese Population

**DOI:** 10.1371/journal.pone.0104525

**Published:** 2014-09-03

**Authors:** Ya-Feng Wen, James Cheng-Chung Wei, Yu-Wen Hsu, Hung-Yi Chiou, Henry Sung-Ching Wong, Ruey-Hong Wong, Shiro Ikegawa, Wei-Chiao Chang

**Affiliations:** 1 Department of Clinical Pharmacy, School of Pharmacy, Taipei Medical University, Taipei, Taiwan; 2 Division of Allergy, Immunology and Rheumatology, Department of Medicine, Chung Shan Medical University Hospital, Taichung, Taiwan; 3 Institute of Medicine, Chung Shan Medical University, Taichung, Taiwan; 4 School of Public Health, Taipei Medical University, Taipei, Taiwan; 5 Department of Public Health, Chung Shan Medical University, Taichung, Taiwan; 6 Department of Pharmacy, Taipei Medical University-Wanfang Hospital, Taipei, Taiwan; 7 Master Program for Clinical Pharmacogenomics and Pharmacoproteomics, School of Pharmacy, Taipei Medical University, Taipei, Taiwan; 8 Laboratory for Bone and Joint Diseases, RIKEN Center for Integrative Medical Science, Yokohama, Japan; Taipei Medicine University, Taiwan

## Abstract

Ankylosing spondylitis (AS) is a highly familial rheumatic disorder and is considered as a chronic inflammatory disease. Genetic factors are involved in the pathogenesis of AS. To identify genes which render people susceptible to AS in a Taiwanese population, we selected six single-nucleotide polymorphisms (SNPs) from previous genome-wide association studies (GWASs) which were associated with AS in European descendants and Han Chinese. To assess whether the six SNPs contributed to AS susceptibility and severity in Taiwanese population, 475 AS patients fulfilling the modified New York Criteria and 527 healthy subjects were recruited. We found that rs10865331 was significantly associated with AS susceptibility and with Bath AS Function Index (BASFI). The AA and AG genotypes of rs10865331 were also significantly associated with a higher erythrocyte sedimentation rate. Our findings provided evidence that rs10865331 is associated AS susceptibility and with disease activity (BASFI) in a Taiwanese population.

## Background

Ankylosing spondylitis (AS) is a systemic, autoimmune disease that causes inflammation of the area where ligaments and tendons insert into the bone, and includes sacroiliitis, spondylitis, spondylodiscitis, and spondylarthritis. The prevalence of AS in Taiwan is about 0.2%∼0.3% which is similar to those in Europe and USA [1], [2]. The male-to-female ratio is about 3∶1 in AS. Twin and family studies have indicated that genetic factors contribute over 90% to the overall AS susceptibility [3], [4]. *Human leucocyte antigen-B 27* (*HLA-B27*) gene is the best-known genetic susceptibility marker for AS, and frequencies of *HLA-B27* are approximately 5.7% and 95% in the general Taiwanese population and Taiwanese AS patients, respectively [5]. Wei et al., showed that HLA-B60 and HLA-B61 are associated with AS in HLA-B27-negative patients in Taiwan [6]. Despite the strong association between HLA and AS, only 1%∼5% of HLA-B27 carriers develop AS [4]. Many non-major histocompatibility complex (non-MHC) regions were found to be significantly associated to AS in genome-wide association studies (GWASs) [7]–[9]. Additionally, genetic polymorphisms of *ORAI1* (rs12313273 and rs7135617) and *STIM1* (rs3750996) were reported to be associated with the pathogenesis of HLA-B27-positive (HLA-B27(+)) AS patients [10], [11].

Previous GWAS reports in European descent indicated that AS development was strongly associated with the 2p15 (rs10865331), 21q22 (rs2242944), *anthrax toxin receptor 2* (*ANTXR2*) (rs4333130), and *interleukin (IL)-23 receptor* (*IL23R*) (rs2310173) loci [7]. John Reveille et al. confirmed the associations of *IL23R* (rs11209026) and *endoplasmic reticulum aminopeptidase 1* (*ERAP1*) (rs27037 and rs27434) with AS [12]. Other significant loci, including *RUNX3* (rs11249215), *LTBR-TNFRSF1A* (rs11616188), and *IL12B* (rs6556416), were also found in a combined discovery and replication study of Europeans [8]. Furthermore, Evans et al., showed a new susceptibility loci of *PTGER4* (rs10440635), *TBKBP1* (10781500), *ANTXR2* (rs4389526), and *CARD9* (rs10781500) that were also involved in the disease pathogenesis. In addition, the *ERAP1* polymorphisms (rs30187) was found to associate with the risk of HLA-B27(+) AS patients [8].

The GWAS conducted in Han Chinese identified new AS susceptibility loci at 6q21 (rs13210693), *HAPLN1-EDIL3* (rs4552569), and *ANO6* (rs17095830). However, there is no strong evidence to indicate the association between *ANTXR2* and *IL23R* polymorphisms and AS in Han Chinese population [9], [13]. Wang *et al.* confirmed the findings of previous studies [14]–[16] that *ERAP1* is a risk factor for AS susceptibility in a Taiwanese population [17]. However, susceptibility loci between *EDIL3*, *HAPLN1* at 5q14.3 and within *ANO6* at 12q12 discovered in a Han Chinese GWAS were negatively associated with Taiwanese population [18], suggesting that genetic factors underlying the susceptibility may differ between these two populations. In this study, we selected SNPs from previous GWAS reports to test whether candidate genetic variations contribute to AS susceptibility and severity in a Taiwanese population.

## Methods

### Subjects

The design of the work was conformed to the *Declaration of Helsinki*. The study was approved by the Institute Review Board of Chung Shan Medical University Hospital. Before any data were collected, we received informed consent from all subjects. All subjects provided a written consent form. AS patients who fulfilled the selection criteria were sequentially solicited at Chung Shan Medical University Hospital in Taichung, Taiwan. The criteria included (a) patients being aged 17∼82 years; (b) cognitive performance not being influenced by other diseases such as dementia; and (c) an AS diagnosis having been made by modified New York criteria developed in 1984. AS diagnosed by qualified rheumatologist. Patients with sacroiliitis were confirmed by a qualified radiologist. The detailed clinical history included extra-spinal manifestations, age on initial symptoms, and laboratory parameters of inflammation, i.e., the erythrocyte sedimentation rate (ESR), and C-reactive protein (CRP). Family history of AS was also recorded. The age of onset of AS symptom was defined as the time when the first symptom (axial symptom, peripheral arthritis, uveitis, or enthesitis) was noted. Peripheral arthritis was defined as the presence of at least one swollen joint. All AS patients in this study had sacroiliitis. These symptoms were recorded in medical record reviews, and were ascertained by a rheumatologist, ophthalmologist, and gastroenterologist.

Potential controls were randomly selected from sequential patients with no significant medical histories or abnormal laboratory results. To exclude controls with potential risks of getting AS, we used the *HLA-B* (rs13202464) polymorphism as a screening factor. Forty-three control subjects carrying the heterozygous AG genotype were excluded. Meanwhile, our data confirmed that *HLA-B* was significantly associated with AS in a Taiwanese population (*P*<0.0001). The Bath AS Disease Activity Index (BASDAI), Bath AS Functional Index (BASFI), and Bath AS Global (BAS-G) were respectively applied to evaluate disease activity, physical function, and global wellbeing. Modified Chinese versions of the BASDAI, BASFI, and BAS-G had good intra-class correlations and Cronbach's alpha values.

### Candidate SNPs

We included SNPs which showed a significant association with AS in three previous GWAS studies (with trend *P*-values of less than or near 10^−7^) [7]–[9] and excluded those hadpublished on Taiwanese populations. The reason for including *ERAP1* (rs27434) is because the other three SNPs on ERAP1 are reported as predisposing factors for AS in Taiwanese [17]; therefore, we test whether SNP, rs27434, has the same effect or not.

### DNA extraction

Blood cells were subjected to DNA extraction by first treating them with 0.5% sodium dodecylsulfate lysis buffer and then protease K (1 mg/ml) to digest nuclear proteins for 4 h at 60°C. Total DNA was harvested using a Gentra (Qiagen, Valencia, CA) extraction kit followed by 70% alcohol precipitation.

### Genotyping

Genotyping for the seven SNPs was carried out using the TaqMan Allelic Discrimination Assay (Applied Biosystems, Foster City, CA). A polymerase chain reaction (PCR) used a 96-well micro-plate with an ABI9700 Thermal Cycler (Applied Biosystems). After the PCR, StepOne software vers. 2.2.2 (Applied Biosystems) was used to detect and analyze the fluorescence. Possession of the *HLA-B27* polymorphism was assessed by flow cytometry as previously described [19].

### Statistical analysis

JMP, Version 8.0 SAS Institute Inc., Cary, NC, 1989–2007 was applied for statistical analyses. Hardy-Weinberg equilibrium (HWE) was used to test the SNPs' allelic frequencies by using χ^2^-test. Statistical differences between the patient and control groups in genotype frequencies were evaluated by χ^2^-test with one degree of freedom or Fisher's exact test. Kruskal-Wallis test was applied to compare the mean of continuous variables (BASDAI, BASFI, and BAS-G). Differences in the values of the ESR, and CRP among AS patients stratified by different genotypes were computed by Wilcoxon rank sum test. Analysis of covariance (ANCOVA) was used to adjust for age, gender, and disease duration. The correlation coefficient was examined between inflammatory biochemical results (ESR and CRP) and BASDI, BASFI, BAS-G. *P*-value of <0.05 was considered statistical significant.

## Results

### Clinical features

Six selected SNPs are shown in Table 1. 475 patients with AS and 527 control subjects were recruited (Table 2). All AS patients were diagnosed according to the modified New York criteria. Their mean age was 39 years; 69.3% were men and 90.7% were HLA-B27 (+). Their mean BASDAI, BASFI and BAS-G scores were 4.3±2.2, 2.1±2.2, and 4.4±2.8, respectively. No significant different distribution of genotypes was found between HapMap CHB population and our control subjects (Table S1 in File S1).

**Table 1:** Six previously reported single nucleotide polymorphisms associated with ankylosing spondylitis.

SNP	Population	Chr.^a^ position	Candidate gene	No. Sample (Case/Control)	*T*rend *p value*	Ref.^b^
rs27434	Han Chinese	5p15	*ERAP1*	1,837/4,231	6.68E-4	9
rs3734523	European	6p22.2	*MHC*	2,053/5,140	1.60E-8	7
rs4672495	European	2p15	-	2,053/5,140	3.30E-9	7
rs10865331	Han Chinese	2p15	-	1,837/4,231	1.98E-8	9
rs11209032	British, Australian	1p31	*IL23R*	4,810/13,579	2.3E-17	8
rs13210693	Han Chinese	6q21	-	1,837/4,231	9.31E-7	9

a . Chr., Chromosome.

b . Reference number (Ref.) is the same as that in the text.

**Table 2:** Characteristics of the Taiwanese population.

Characteristic	AS patient	Control
Number of subjects	475	527
Gender: male No (%)	323 (68.0)	365 (69.3)
Age: mean ± SD (years)	39.0±11.3	39.0±11.9
BASDAI	4.3±2.2	
BASFI	2.1±2.2	
BAS-G	4.4±2.8	

### rs10865331 is associated with AS susceptibility

All genotype distributions of the polymorphisms fulfilled the criteria of HWE (*P*>0.05). Table 3 showed a comparison between AS cases and controls for each genotype and individual allele for all six SNPs. The rs10865331 A allele carrier (contained AA and AG genotypes) had 1.65 fold risk compared with GG genotype carrier (OR (95% CI) = 1.65 (1.21–2.23), *P*-value = 0.001). G allele of rs13210693 at 6q21, had a significant correlation with AS susceptibility (OR (95% CI) = 1.38 (1.96–2.06), *P*-value = 0.039). Since *P*-value was considered significant when it was less than 0.0083, after applying Bonferroni correction, only rs10865331 polymorphism showed significant association with AS.

**Table 3:** Association risk SNPs in previous study and current study.

		European cohort^7^	British, Australia cohort^8^	Han Chinese cohort^9^	Current study			
SNP	Minor allele	Allele frequency^a^	Allele frequency^a^	Allele frequency^a^	Allele frequency^a^	OR (95% CI)^b^	*P*-value^b^	HWE^c^
rs3734523	A	0.09/0.12			0.02/0.04	0.58 (0.33–1.00)	0.052	0.265
rs4672495	G	0.36/0.32			0.17/0.19	0.89 (0.67–1.17)	0.400	0.632
rs10865331	A	0.43/0.36	0.45/0.37	0.54/0.48	0.53/0.47	1.65 (1.21–2.23)	**0.001** **	0.817
rs11209032	G		0.36/0.33		0.49/0.49	1.04 (0.26–1.96)	0.794	0.073
rs27434	G	0.26/0.21		0.55/0.53	0.46/0.50	0.86 (0.94–1.96)	0.347	0.336
rs13210693	G			0.47/0.44	0.52/0.48	1.38 (1.96–2.06)	0.039	0.867

χ^2^-test was applied for testing genotype frequencies of SNPs in controls and patients with AS.

**Significant (p<0.0017) value is in bold.

a Allele frequency in case/control.

b The OR and *P*-value were showed for dominant model of SNPs.

c HWE were performed by chi-square.

7,8,9 The reference numbers are as the same as that in the text.

### rs10865331 is associated with AS severity

**Figure 1:**
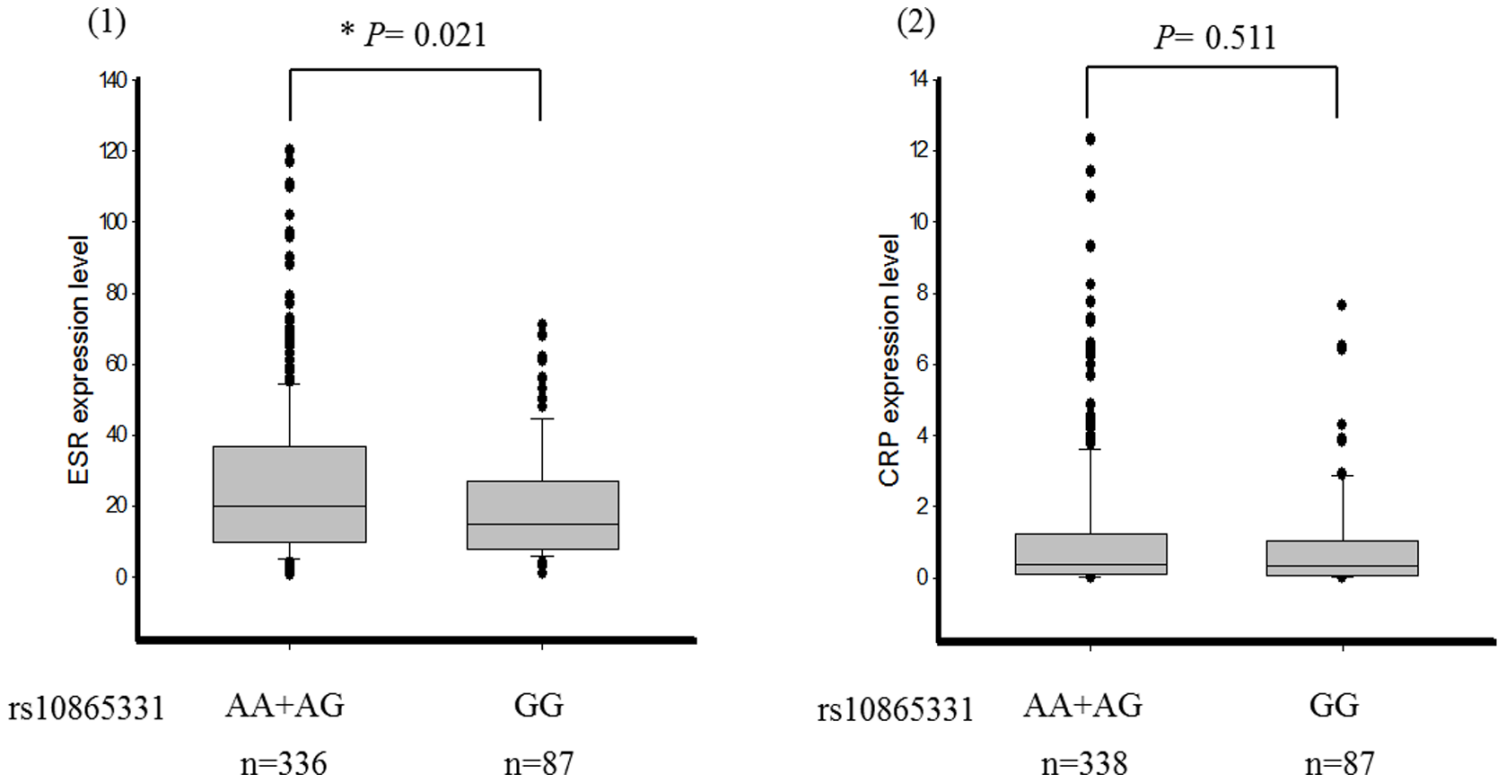
Comparison of the erythrocyte sedimentation rate (ESR) and C-reactive protein (CRP) levels among different genotypes of rs10865331 in ankylosing spondylitis (AS) patients.

**Figure 2:**
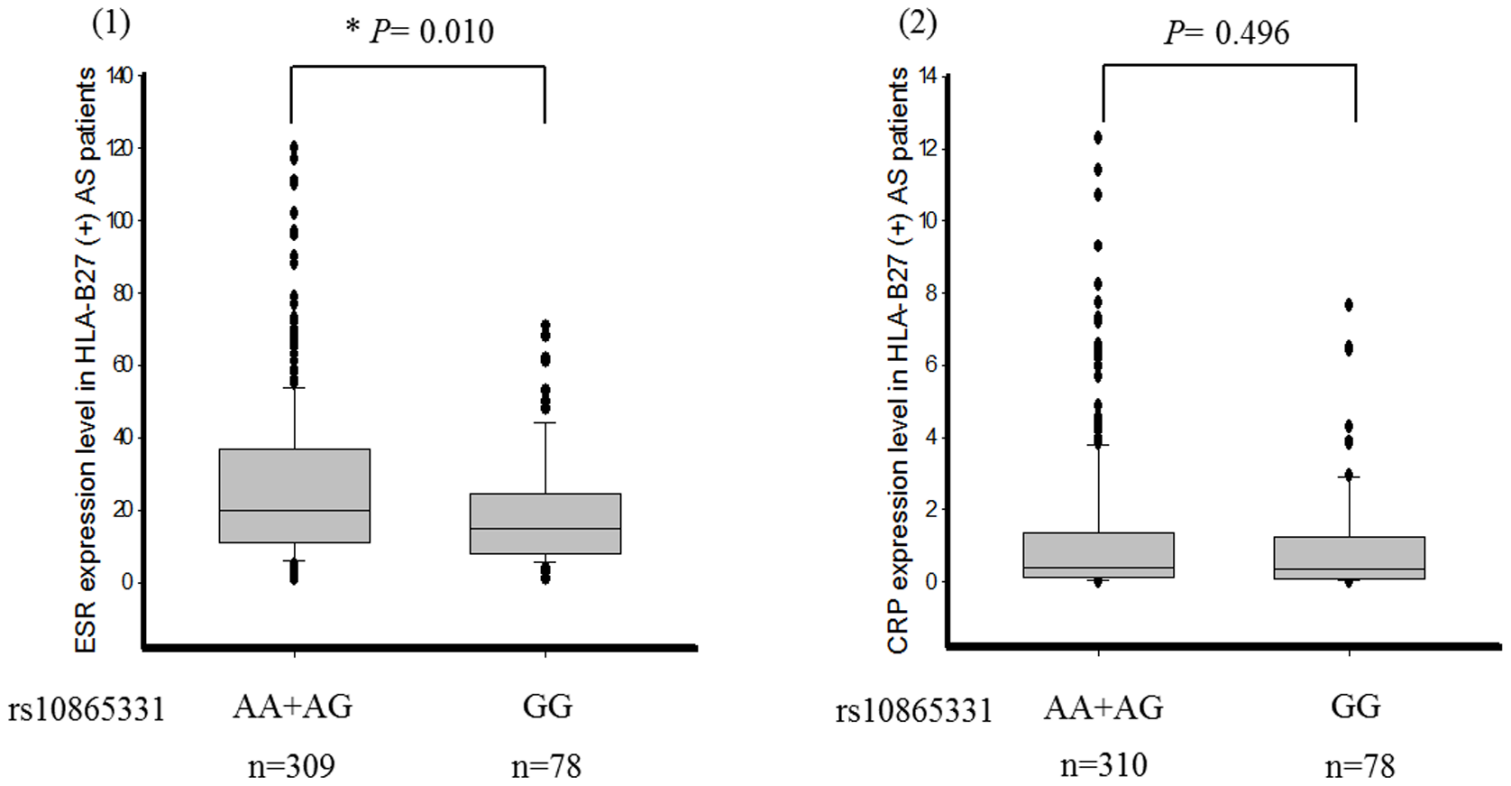
Comparison of the erythrocyte sedimentation rate (ESR) and C-reactive protein (CRP) levels among different genotypes of rs10865331 in ankylosing spondylitis (AS) patients positive for HLA-B27.

We investigated the relationship between genetic polymorphisms and clinical phenotypes including the BASDAI, BASFI, and BAS-G. We found that rs10865331 was highly associated with the BASFI (*P*-value = 0.033). However, the results showed no statistical significance after applying the Bonferroni correction and adjusting for gender and disease duration (Table 4). We further analyzed the association between inflammatory biochemical results (ESR and CRP) and the rs10865331 genetic polymorphism. rs10865331 AA and AG genotypes were significantly correlated with an increased ESR compared to the GG genotype in AS patients (*P*-value = 0.021) (Figure 1). The means±standard deviations of the ESR for the combined AA and AG genotypes and GG genotype were 26.00±21.74 and 20.07±16.26, respectively. However, the risk allele (A allele) of rs10865331 showed no correlation with CRP (*P*-value  = 0.511). CRP values were 1.17±1.89 for the combined AA and AG genotypes and 0.96±1.49 for the GG genotype. In addition, the A allele of rs10865331 in HLA-B27(+) AS patients had a significant association with the ESR (*P*-value = 0.010) (Figure 2).

**Table 4:** Difference in disease activity scores in the AS patients stratified by genotypes.

SNP	Genotype	BASDAI	BASFI	BAS-G
rs3734523	AA	3.84	4.01	5.00
	AG	3.61±2.48	1.35±2.23	3.79±3.07
	GG	4.32±2.19	2.03±2.16	4.41 ± 2.73
Unadjusted *p* value		0.289	0.170	0.538
Adjusted *p* value		0.361†	0.194§	0.573†
rs4672495	GG	3.99±2.63	0.79±0.82	4.13 ± 2.80
	GT	4.22±2.31	2.06±2.25	4.31 ± 2.76
	TT	4.32±2.15	2.08±2.24	4.42 ± 2.76
Unadjusted *p* value		0.847	0.210	0.862
Adjusted *p* value		0.737†	0.275§	0.888†
rs10865331	AA	4.66±2.09	2.35±2.34	4.66 ± 2.73
	AG	4.16±2.22	2.13±2.32	4.31 ± 2.75
	GG	4.22±2.25	1.47±1.69	4.22 ± 2.68
Unadjusted *p* value		0.132	0.033*	0.391
Adjusted *p* value		0.104†	0.133§	0.500†
rs11209032	AA	4.61±2.10	2.11±2.36	4.56 ± 2.55
	AG	4.09±2.10	1.97±2.12	4.32 ± 2.77
	GG	4.34±2.44	2.12±2.32	4.36 ± 2.90
Unadjusted *p* value		0.096	0.941	0.604
Adjusted *p* value		0.156†	0.467§	0.737†
rs27434	GG	4.56±2.12	2.05±2.08	4.53 ± 2.50
	AG	4.17±2.28	2.03±2.14	4.50 ± 2.85
	AA	4.33±2.11	1.61±1.92	3.84 ± 2.60
Unadjusted *p* value		0.593	0.364	0.083
Adjusted *p* value		0.407†	0.331§	0.125†
rs13210693	AA	4.37±2.30	1.93±2.20	4.10 ± 2.79
	AG	4.18±2.20	1.95±2.21	4.26 ± 2.66
	GG	4.60±2.07	2.28±2.09	4.83±2.75
Unadjusted *p* value		0.203	0.209	0.127
Adjusted *p* value		0.271†	0.368§	0.100†

BAS, Bath Ankylosing Spondylitis; DAI, Disease Activity Index; FI, Function Index; G, Global. Data are presented as the mean ± standard deviation. The Kruskal-Wallis test is applied to exam the difference in disease activity scores in the AS patients stratified by genotypes.

† Adjusted for the effects of age and gender.

§ Adjusted for the effects of age, gender, and disease duration. Significant (*p*<0.05) values are in bold.

### ESR and CRP showed positive correlations with the Bath indices

**Figure 3:**
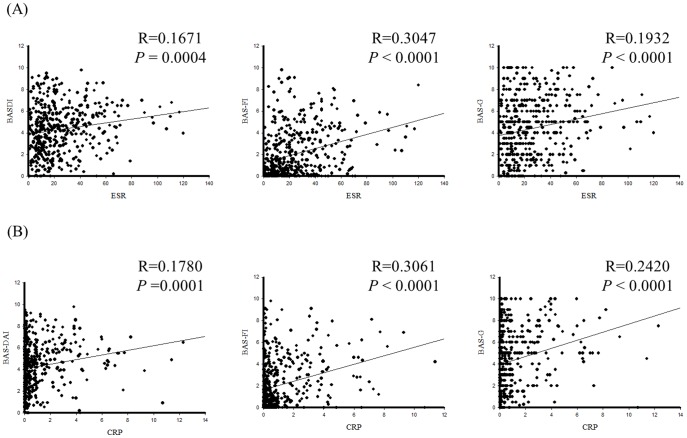
Correlation analysis between erythrocyte sedimentation rate (ESR), C-reactive protein (CRP) and Bath Indices (BASDI, BASFI and BAS-G).

Table 4 indicated the relationship between rs10865331 and BASFI. We, therefore, further tested the correlation between inflammatory biochemical data (ESR and CRP) and disease activity (BASDI, BASFI, BAS-G). As shown in Figure 3A, ESR associated with BASDI (R = 0.1671, *P*-value = 0.0004), BASFI (R = 0.3047, *P*-value<0.0001), and BAS-G (R = 0.1932, *P*-value<0.0001). Regarding to CRP, positive correlations between CRP and BASDI (R = 0.178, *P*-value<0.0001), BASFI (R = 0.3061, *P*-value<0.0001), BAS-G (R = 0.2420, *P*-value<0.0001) were found. (Figure 3B)

## Discussion

In the case-control study, rs10865331 was significantly associated with a genetic predisposition for AS and also affected patients' daily functional activities represented by BASFI. However, the association between AS and the BASFI became insignificance after adjusting for age, gender, and the duration of the disease, this is possibly caused by the variation of scores among patients. We, therefore, used clinically important measures, including the ESR and CRP, to support the correlation between candidate SNPs and disease severity. We found an association between inflammatory biochemical lab data, i.e., the ESR, and disease severity in AS patients. After excluding HLA-B27(−) AS patients, the same trend was also found. This is the first study to confirm that the intergenic SNP, rs10865331, on chromosome 2p15 is highly associated with AS disease in a Taiwanese population.

The intergenic region, 2p15, of the associated rs10865331 SNP was replicated in several studies of Spanish, Korean and Han Chinese populations [14], [15], [20]. The rs10865331 polymorphism located 99 kb upstream of *B3GNT2* and 182 kb downstream of *TMEM17*. Although the mechanism and functions leading to progression of AS still need to be elucidated, an SNP on *B3GNT2* was found to be a susceptibility marker for rheumatoid arthritis in a Japanese GWAS meta-analysis [21]. In addition, a polymorphism (rs6545946) at 2p15 could be a plausible candidate SNP for Crohn's disease, which is a common comorbid condition of AS in Ashkenazi Jewish patients [22]. The evidence implies that intergenic regions of chromosomes 2p15 are somehow associated with certain immune diseases.

*ERAP1* involves peptide trimming as presented by HLA class I molecules within the endoplasmic reticulum and only affects AS risk in HLA-B(+) individuals [23]. *ERAP1* may play an important role in the shedding of proinflammatory cytokine receptors and downregulation of inflammatory responses [24]. Nonetheless, the association between *ERAP1* polymorphisms and AS is divergent in different ethnic groups in genetic studies. In a meta-analysis study, rs27434 showed no significant correlation with AS, which corresponded with our results [16]. Wang et al. identified another *ERAP1* SNP, rs27037, which can predict AS susceptibility and syndesmophyte formation [17]. rs27434 is a synonymous SNP, and rs27037 is located in the intron possibly affecting the regulation of MHC I heavy chain homodimers leading to unfolded protein responses [25]. This may explain the different effects of the two SNPs.

In 2013, evidence from high-density genotyping of immune-related loci indicated that major histocompatibility complex (MHC) class I presentation and IL-23 pathway are key elements in the development of ankylosing spondylitis [26]. Indeed, previous studies also confirmed an important role of *IL23R* gene in AS [27]. IL23R belongs to the hemopoietin receptor family for IL-23, a proinflammatory cytokine, and is involved in the production and differentiation of memory T-cells [27]. rs11209032 showed a significant correlation with AS in a UK case-control study and meta-analysis [28]. Another meta-analysis showed that rs11209032 was significantly associated with AS in European but not Asian populations [29]. Genetic differences between different ethnic groups may explain why rs11209032 had no statistical association with AS in a Taiwanese population. Interestingly, many studies reported that variations in *IL23R* were not associated with AS [15], [30] but were correlated with inflammatory bowel disease (IBD) which is clinically related to AS [31]. Our previous results also indicated that two susceptibility SNPs identified from Han Chinese GWAS study [9], *HAPLN1-EDIL3* (rs4552569) and *ANO6* (rs17095830), were not associated with AS severity but IBD in a Taiwanese population [18]. Further studies are needed to clarify the mechanism by which MHC and IL-23 involve the pathogenesis of AS and IBD.

By a systemic overview of genetic variations of the risk in three previous GWASs, we were better able to understand the high-risk polymorphisms in the Taiwanese population. A new susceptibility polymorphism at 2p15 (rs10865331) was associated with AS and disease severity. However, even with large-scale screening of candidate susceptibility loci, the exact pathological mechanism and interactions between AS genes, e.g., *ERAP1* and *HLA-B*, are still unknown. Furthermore, examination of epigenetic factors and copy number variations are required, and functional studies that show how susceptible genes actually affect AS should be conducted. Combining all the functional causal alleles in Taiwanese populations may improve our understanding of the genetic basis of the disease and lead to novel treatment approaches.

## Supporting Information

File S1 **Supporting tables.** Table S1, Comparison between HapMap CHB population and Taiwanese population. Table S2, Genotype and allele frequencies in controls and patients among HLA-B27 (+) with AS.
(DOCX)
