# Supplementary material for: rs10865331 Associated with Susceptibility and Disease Severity of Ankylosing Spondylitis in a Taiwanese Population
Source: PLoS One. 2014 Sep 3;9(9):e104525. doi: 10.1371/journal.pone.0104525 (PMC4153545; doi:10.1371/journal.pone.0104525)
Supplement: File S1 — Supporting tables. Table S1, Comparison between HapMap CHB population and Taiwanese population. Table S2, Genotype and allele frequencies in controls and patients among HLA-B27 (+) with AS. (DOCX) [file pone.0104525.s001.docx]

**Supplementary section**

| **Table S1.** Comparison between HapMap CHB population and Taiwanese population. | | | | |
| --- | --- | --- | --- | --- |
| SNP | Genotype | HapMap CHB population n.(%) | Current study control  n (%) | *P*-value |
| rs3734523 | AA | 0 (0) | 1 (0.2) | 0.569† |
|  | AG | 7 (5.1) | 36 (7.5) |  |
|  | GG | 130 (94.9) | 444 (92.3) |  |
| rs4672495 | GG | 2 (1.5) | 20 (4.2) | 0.364† |
|  | GT | 42 (30.7) | 142 (29.6) |  |
|  | TT | 93 (67.9) | 318 (66.2) |  |
| rs10865331 | AA | 25 (18.2) | 112 (23.9) | 0.382 |
|  | AG | 68 (49.6) | 216 (46.1) |  |
|  | GG | 44 (32.1) | 141 (30.0) |  |
| rs11209032 | GG | 32 (23.4) | 121 (25.0) | 0.308 |
|  | AG | 74 (54.0) | 228 (47.0) |  |
|  | AA | 31 (22.6) | 136 (28.0) |  |
| rs27434 | GG | 31 (23.0) | 89 (24.2) | 0.893 |
|  | AG | 72 (53.3) | 187 (51.0) |  |
|  | AA | 32 (23.7) | 91 (24.8) |  |
| rs13210693 | GG | 34 (24.8) | 109 (24.5) | 0.921 |
|  | AG | 62 (45.3) | 209 (47.1) |  |
|  | AA | 41 (29.9) | 126 (28.4) |  |
| † Fisher’s exact test was applied. | | | | |

| **Table S2.** Genotype and allele frequencies in controls and patients among HLA-B27 (+) with AS | | | | | | | |
| --- | --- | --- | --- | --- | --- | --- | --- |
| SNP | Minor allele | Genotypes ^a^ | | Allele frequency ^b^ | OR (95% CI) ^c^ | *P*-value ^c^ | HWE ^d^ |
|  |  | Cases | Controls |  |  |  |  |
| rs3734523 | A | 1/18/377 | 1/36/444 | 0.03/0.04 | 0.60 (0.34-1.06) | 0.081 | 0.272 |
| rs4672495 | G | 12/118/274 | 20/142/318 | 0.18/0.19 | 0.93 (0.70-1.23) | 0.621 | 0.603 |
| rs10865331 | A | 101/218/82 | 112/216/141 | 0.52/0.47 | 1.67 (1.23-2.28) | **0.001**** | 0.949 |
| rs11209032 | G | 107/194/100 | 121/228/136 | 0.51/0.49 | 1.17 (0.87-1.58) | 0.298 | 0.159 |
| rs27434 | G | 71/190/96 | 89/187/91 | 0.47/0.50 | 0.90 (0.64-1.25) | 0.520 | 0.249 |
| rs13210693 | G | 89/212/101 | 109/209/126 | 0.49/0.48 | 1.18 (0.87-1.60) | 0.286 | 0.918 |
| χ^2^-test was applied for testing genotype frequencies of SNPs in controls and patients with AS. **Significant (p<0.0017) value is in bold. ^a^ Genotype number of minor allele homozygotes/heterozygotes/major allele homozygotes. ^b^ Allele frequency in case/control. ^c^ The OR and *P*-value were showed for dominant model of SNPs. ^d^ HWE were performed by chi-square. | | | | | | | |
